# CHD4 in the DNA-damage response and cell cycle progression: not so NuRDy now

**DOI:** 10.1042/BST20130027

**Published:** 2013-05-23

**Authors:** Aoife O’Shaughnessy, Brian Hendrich

**Affiliations:** Wellcome Trust - Medical Research Council Stem Cell Institute and Department of Biochemistry, Tennis Court Road, University of Cambridge, Cambridge CB2 1QR, U.K.

**Keywords:** cell cycle, chromatin, chromodomain helicase DNA-binding 4 (CHD4), DNA damage, DNA repair, nucleosome remodelling and deacetylation (NuRD), ATM, ataxia telangiectasia mutated, ATR, ataxia telangiectasia- and Rad3-related, BRCA1, breast cancer early-onset 1, CHD, chromodomain-helicase-DNA-binding, DDR, DNA-damage response, EP300, E1A binding protein p300, γH2AX, phosphorylated histone H2AX, HDAC, histone deacetylase, MDC1, mediator of DNA damage checkpoint 1, MTA, metastasis-associated protein, NuRD, nucleosome remodelling and deacetylation, PARP, poly(ADP-ribose) polymerase, PHD, plant homeodomain, RNF, RING finger protein

## Abstract

The CHD4 (chromodomain-helicase-DNA-binding 4) (or Mi-2β) protein is a founding component of the NuRD (nucleosome remodelling and deacetylation) complex. NuRD has long been known to function in transcriptional regulation, and is conserved throughout the animal and plant kingdoms. In recent years, evidence has steadily accumulated indicating that CHD4 can both function outside of the NuRD complex and also play important roles in cellular processes other than transcriptional regulation. A number of loss-of-function studies have identified important roles for CHD4 in the DNA-damage response and in cell cycle progression through S-phase and into G_2_. Furthermore, as part of NuRD, it participates in regulating acetylation levels of p53, thereby indirectly regulating the G_1_/S cell cycle checkpoint. Although CHD4 has a somewhat complicated relationship with the cell cycle, recent evidence indicates that CHD4 may exert some tumour-suppressor functions in human carcinogenesis. CHD4 is a defining member of the NuRD complex, but evidence is accumulating that CHD4 also plays important NuRD-independent roles in the DNA-damage response and cell cycle progression, as well as in transcriptional regulation.

## Introduction

The CHD3 (chromodomain-helicase-DNA-binding 3) (or Mi-2α) and CHD4 (or Mi-2β) proteins were originally identified as autoantigens in dermatomyositis, a connective tissue disease, which imparts an increased risk of malignancy [1–3]. These proteins are widely conserved throughout the animal and plant kingdoms, but are absent from yeast [4]. CHD family proteins are members of the SNF2 superfamily of ATPases, which use the energy derived from ATP hydrolysis to remodel nucleosome structure [5,6]. Specifically, the ATPase/helicase domain of CHD4 is able to bind and mobilize nucleosomes along DNA [7].

In addition to an ATPase domain, CHD3 and CHD4 both contain two PHD (plant homeodomain) fingers and two chromodomains. The presence of the PHD fingers distinguishes the CHD3/CHD4 subfamily from the rest of the CHD family [8]. The role each domain plays in the context of the full-length mammalian protein is not completely understood. Although many chromodomains have been shown to act as histone-binding modules, the chromodomains of the *Drosophila melanogaster* dMi-2 orthologue have been shown to bind both DNA and nucleosomes [9]. The PHD fingers in CHD4, when expressed alone, are able to bind histone H3 with a preference for unmodified Lys^4^ (H3K4) and trimethylated Lys^9^ (H3K9me3) [10–13]. This is consistent with evidence that intact CHD4-containing protein complexes can be isolated from crude nuclear extract via affinity with unmethylated H3K4 [14–17]. In the presence of the chromodomains and ATPase domain, however, the PHD fingers appear to preferentially modify the activity of, and physically interact with, the ATPase domain in *in vitro* experiments [10,12]. These observations raise the intriguing possibility that the ATPase activity of CHD4 may be indirectly regulated by the binding of histone H3.

CHD4 is a core component of the NuRD (nucleosome remodelling and deacetylation) complex. NuRD is an abundant co-repressor complex with a broad cellular and tissue distribution and a variable subunit composition [4]. NuRD couples chromatin remodelling activity, provided by the ATPase of Mi-2β, with protein deacetylation via the associated HDAC1 (histone deacetylase 1) and HDAC2 proteins in one multisubunit complex [18–21]. The central function of the NuRD complex has long been considered to be the creation of a chromatin environment that is refractory to active transcription [22]. However, it is becoming increasingly apparent that so-called ‘transcriptional repressors’ such as the NuRD complex exert more complex influences over transcription than straightforward silencing [23]. For example, NuRD has been implicated in transcriptional fine-tuning, which is necessary to drive developmental processes in mouse ES (embryonic stem) cells [24]. Although CHD4 is a defining NuRD component, there has been a steady accumulation of evidence that it also functions independently of NuRD [23,25,26]. In recent years, it has emerged further that CHD4 plays important roles in DDR (DNA-damage response) and cell cycle progression. In the present article, we summarize the evidence that this well-characterized transcriptional regulator also influences DNA repair and cell cycle in mammalian cells.

## CHD4/NuRD and the DDR

Chromatin-remodelling proteins have long been implicated in the DDR. Compacted chromatin is inhibitory to full activation of the DDR and a considerable amount of evidence exists supporting a role for chromatin-remodelling factors in relieving this inhibition [27–30]. Very shortly after the initial descriptions of NuRD, CHD4 and another NuRD component protein, HDAC2, were found to interact with the protein kinase ATR (ataxia telangiectasia- and Rad3-related) [31]; however, it is not clear whether either of these proteins interact with ATR as part of NuRD or independently of it. More recently, a number of groups reported that CHD4 is a phosphorylation target of the DDR kinases ATR and ATM (ataxia telangiectasia mutated) [32–34], and that its expression is induced by UV irradiation [35]. Therefore it was clear that CHD4, and possibly NuRD, can interact directly with different aspects of the DDR.

Further evidence for a link between NuRD and the DDR came from a study demonstrating that some NuRD components show reduced expression in the premature aging disease Hutchinson–Gilford progeria syndrome and also as a consequence of normal aging [36]. Among the defects observed in both aged cells and after knockdown of some NuRD components [including HDAC1, MTA3 (metastasis-associated protein 3) and CHD4], was an increase in γH2AX (phosphorylated histone H2AX), a marker of DNA damage [36], consistent with CHD4 and possibly NuRD playing some role in protecting the integrity of genomic DNA. Once again, most of the NuRD components analysed are not exclusively found in NuRD, so its involvement remained uncertain.

**Figure 1:**
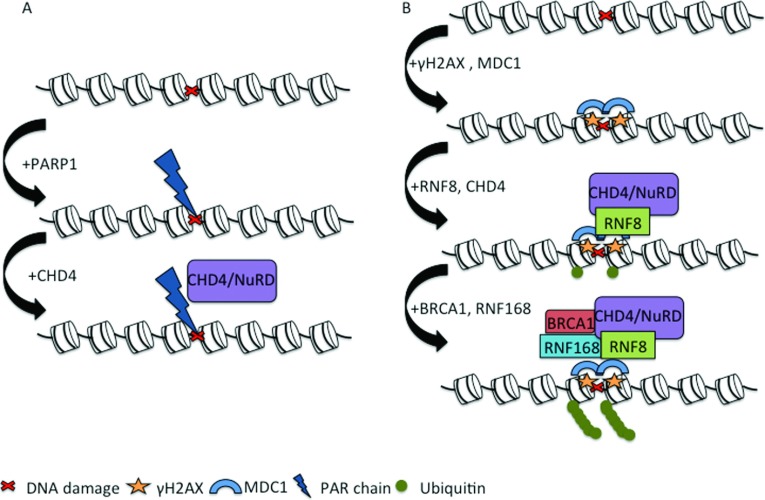
CHD4 recruitment to sites of DNA damage (**A**) CHD4 can be recruited to sites of DNA damage in a PARP-dependent manner, after which it can mediate the recruitment of NuRD complex proteins. PAR, poly(ADP-ribose). (**B**) CHD4 can also interact with RNF8 and the two proteins co-operate to create a chromatin environment permissive for the amplification of the DNA-damage repair signal and thus recruitment of downstream repair factors such as the ubiquitin ligase RNF168 and BRCA1.

In the last three years, a number of studies have firmly established the role of CHD4, and possibly NuRD, in mediating the DDR. CHD4 can be recruited to sites of DNA damage via two distinct mechanisms [37,38]. First, CHD4 can be recruited to sites of DNA damage by binding to poly(ADP-ribosyl)ated proteins, including PARP1 [poly(ADP-ribose) polymerase 1] [38] (Figure 1A). This event in turn mediates the PARP-dependent recruitment of the NuRD component proteins HDAC1 and MTA2. Convincingly, PARP inhibition was found to abrogate CHD4/NuRD accumulation at sites of DNA damage [38]. Whereas this study confirmed the previous observation that CHD4 is a phosphorylation target of ATM, it also showed that this phosphorylation did not influence the function that it plays in the DDR [38].

The second manner in which CHD4 is recruited to sites of DNA damage is via interaction with the ubiquitin ligase RNF (RING finger protein) 8 (Figure 1B). RNF8 is initially recruited to sites of DNA damage via its affinity for MDC1 (mediator of DNA damage checkpoint 1), which binds to γH2AX [39]. DDR must take place within the chromatin template and a well-characterized chromatin change brought about by the DDR is the phosphorylation of the histone variant H2AX (forming γH2AX) at sites of DNA damage [40]. Upon recruitment by RNF8, the chromatinremodelling activity of CHD4 is proposed to decondense the chromatin at the DNA-damage site, which stimulates the formation of ubiquitin conjugates by both RNF8 and another ubiquitin ligase, RNF168 [37]. The stimulation of ubiquitylation activity of RNF8/RNF168 is a necessary prerequisite for amplification of the DNA-damage repair signal and recruitment of downstream DNA-damage repair proteins [41]. Conclusively, knockdown of *CHD4* results in reduced ubiquitylation at DNA double-strand breaks as well as a corresponding reduction in the accumulation of the repair proteins RNF168 and BRCA1 (breast cancer early-onset 1), highlighting its key role in amplifying the DDR once recruited by RNF8 [37]. Intriguingly, however, tethering RNF8 to chromatin appears to bypass the requirement for CHD4 [37]. Thus it appears that CHD4 facilitates the access of RNF8 to sites of DNA damage, most likely by creating a local chromatin environment that is permissive to the assembly of checkpoint and repair machineries at DNA lesions.

## CHD4 and DNA damage: cause and/or cure?

A number of studies have implicated chromatin-remodelling complexes in the maintenance of genomic integrity [42–44]. Whereas many studies have now examined the role of CHD4/NuRD in the DDR, one important unresolved issue is whether CHD4 normally functions to maintain genome integrity, or is simply required to repair damage caused by environmental or replicative stresses. A number of studies have now reported that depletion of CHD4 in mammalian cells results in an increase in markers of replication stress and hypersensitivity to ionizing radiation, resulting in an increased load of spontaneous damage [41,45,46]. One study found further that knockdown of MTA2, a NuRD component protein, led to an increased sensitivity to ionizing radiation, providing evidence that NuRD also participates in DNA repair [41]. Notably, a mutant version of CHD4 which lacks helicase activity was found to be unable to rescue the depletion phenotype, demonstrating the importance of chromatin remodelling in this aspect of CHD4 function [41]. The increase in levels of markers of replicative stress, and the occurrence of spontaneous DNA damage upon depletion of CHD4 in mammalian cells is consistent with CHD4 playing an important role in the maintenance of genomic integrity. These studies further implicate NuRD in the DNA-repair process, although whether NuRD also plays a role in genome stability is less clear.

## CHD4/NuRD and control of cell cycle progression

**Figure 2:**
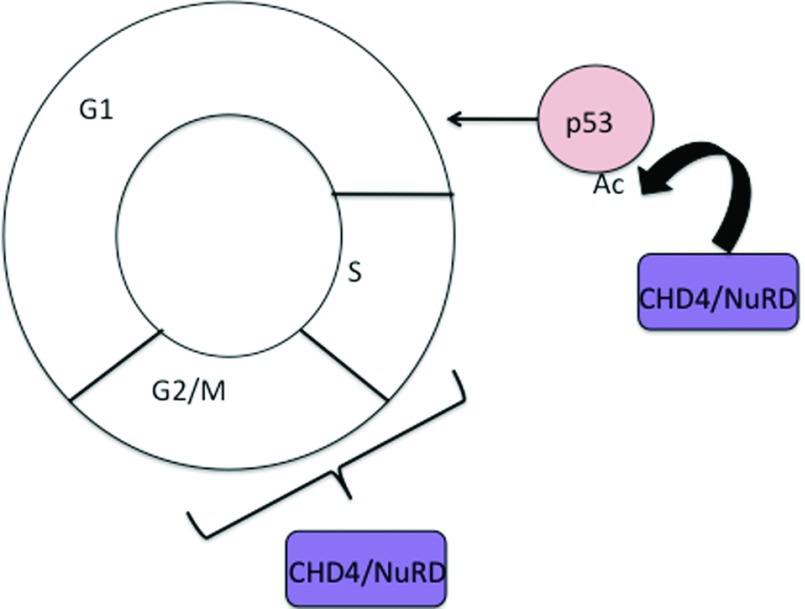
CHD4/NuRD contribution to cell cycle control NuRD deacetylates p53 thus reducing p53 activity and driving cells through the G_1_/S transition. CHD4 loss has also been shown to have an effect on both the S- and G_2_-phases of the cell cycle, presumably via its role in the DDR.

In addition to functioning in the DDR, and potentially acting to maintain chromatin structure, CHD4 has also been shown to contribute to the control of cell cycle progression (Figure 2). CHD4/NuRD has emerged as an important regulator of the G_1_/S transition through its control of p53 deacetylation [38,47]. NuRD-mediated deacetylation restricts the activity of p53, thereby facilitating progression through the R-point at the G_1_/S boundary. Upon depletion of CHD4, p53 becomes hyperacetylated and hyperactive, which in turn leads to increased p21 expression and ultimately G_1_/S arrest [38]. Interestingly, knockdown of the lysine acetyltransferase EP300 (E1A-binding protein p300) can rescue the cell cycle defects in CHD4-depleted cells, consistent with a model in which EP300 and NuRD together control the acetylation levels of p53 [38].

The control exerted by CHD4/NuRD on cell cycle progression is not, however, straightforward. In addition to its role in antagonizing p53 and driving cells through the G_1_/S transition, its function in DNA-damage repair is also required for successful completion of S-phase and progression through the G_2_/M checkpoint, as cells depleted of *CHD4* show delayed progression through S-phase and accumulation in G_2_ [41,45,46]. Curiously, however, genetic analyses of the functions of NuRD components in mammalian cells have yet to identify any that are required for cell viability [25,48–54]. In contrast, no actively cycling cell population lacking *CHD4* has yet been reported. Thus it may well be that CHD4 exerts its crucial cell cycle regulation independently of NuRD, although genetic redundancy among genes encoding NuRD component proteins may yet mask an essential cellular function for NuRD.

## CHD4 and cancer?

Whereas a number of NuRD complex subunits have been implicated in cancer development [55], *CHD4* has not emerged as a tumour suppressor from genetic analyses in humans or mice. This could perhaps be due to the complicated relationship that CHD4 has with the cell cycle. As part of NuRD, CHD4 acts to restrict p53 activity via deacetylation [38,47]. This puts CHD4/NuRD in direct opposition to the tumour-suppressor activities of p53 and p21. However, in its role as a DNA-repair protein, CHD4 functions as a tumour suppressor alongside proteins such as BRCA1. Thus the consequences of *CHD4* mutation on the cell cycle and tumorigenesis might not be straightforward.

Despite this complex relationship with the cell cycle, recent evidence has indicated that CHD4 may play a tumour-suppressor function in some tumour types. *CHD4* expression was reported to be reduced in gastric and colorectal cancers with microsatellite instability [56], as is often seen for classic tumour-suppressor genes. More recently, large-scale exome sequencing revealed that 17% of endometrial cancers analysed showed somatic mutations in *CHD4* [57]. Notably, mutations affecting the ATPase domain accounted for half of all mutations identified. Although the overall architecture of the NuRD complex is far from clear, perhaps its ATPase activity, encoded in *CHD4*, is not important for the complex's protein deacetylation activity [37,38]. Hence a missense mutation in the *CHD4* ATPase domain might specifically target the tumour-suppressing DNA-repair activity of *CHD4* without compromising the p53-antagonizing functions of NuRD.

## Conclusions and remaining questions

Overall the role of CHD4 in DNA repair and cell cycle progression has become increasingly well described over the last few years. It makes intuitive sense that chromatin remodelling should have some part to play in these biological processes; however, the specific contributions of CHD4 and of NuRD are only now being examined in detail. This is somewhat surprising given that the first publications on the NuRD complex indicated that it could be involved in the DDR [31].

Two distinct mechanisms have been proposed by which CHD4 is recruited to sites of DNA damage (Figure 1). One group proposed that CHD4 recruitment is through an association with the ubiquitin ligase protein RNF8, and that together with RNF8 CHD4 plays a crucial role in the amplification of the DDR through the creation of a permissive chromatin environment [37]. However, the finding that CHD4 accumulation at DNA-damage sites is not impaired in H2AX-deficient cells casts some doubt on this model, as RNF8 is recruited to sites of DNA damage through an interaction with MDC1 and γH2AX [38]. A second proposed mechanism of recruitment is via binding of PAR chains, which are deposited on chromatin at sites of DNA damage by PARP proteins. While one study reported that PARP inhibition abrogated the accumulation of CHD4 at DNA-damage sites [38], another reported that this had no influence on CHD4 recruitment [37]. Perhaps, as suggested by the authors of the latter study, these two potential mechanisms represent two distinct and unrelated modes of potential recruitment of CHD4 to sites of DNA damage [37].

Another area upon which the influence of CHD4/NuRD is not yet clear is the maintenance of chromatin structure and, by extension, the maintenance of genomic integrity. Upon CHD4 depletion, there appears to be an increased load of spontaneous DNA damage, as well as loss of the heterochromatic histone modification H3K9me3 and up-regulation of markers of replication stress such as RAD51 [36,41,45,46,58]. Certainly these findings indicate that CHD4 may have a genome-protective role; however, it remains to be seen whether this role is in the replication process and indeed whether this would constitute the prevention of DNA damage or simply the repair of stress-induced damage [46].

Finally, CHD4 and NuRD have also been demonstrated to function in cell cycle progression. The most clear-cut example of this is NuRD's function in controlling p53 acetylation levels. NuRD deacetylates p53, interfering with its activity [38,47]. Intriguingly, the acetylation status of p53 and progression through the G_1_/S cell cycle boundary appears to be controlled by interplay between the antagonistic deacetylation and acetylation activities of NuRD and EP300 respectively [38]. In addition to an effect at the G_1_/S boundary, loss of CHD4 function has also been reported to lead to an S-phase delay and even an accumulation at the G_2_/M checkpoint [41,46]. As yet, no adverse effect of NuRD complex disruption on cell viability has been reported. In contrast, CHD4 depletion in a human cell line resulted in apoptosis [41], possibly indicating that CHD4 exerts a crucial cell-cycle-related function independently of the NuRD complex.

Given the evidence for CHD4 function in DNA repair and cell cycle progression, it is perhaps unsurprising that it has also recently been implicated in cancer. The fact that CHD4/NuRD appears to function in an oncogenic fashion in some instances (e.g. p53 deacetylation), whereas CHD4 exerts a tumour-suppressor function in others (e.g. through amplification of the DDR) may mean that deletions or amplifications of key component genes may have different effects in different tumour types. Nonetheless, given the crucial roles played by CHD4 not only in transcriptional regulation, but also in DNA repair, cell cycle progression and the maintenance of genomic integrity, it seems likely that the above examples of CHD4 being involved in cancer will be the first of many.
